# TNFα-Signaling Modulates the Kinase Activity of Human Effector Treg and Regulates IL-17A Expression

**DOI:** 10.3389/fimmu.2019.03047

**Published:** 2020-01-21

**Authors:** Paulo C. M. Urbano, Xuehui He, Bennie van Heeswijk, Omar P. S. Filho, Henk Tijssen, Ruben L. Smeets, Irma Joosten, Hans J. P. M. Koenen

**Affiliations:** ^1^Laboratory Medical Immunology, Department of Laboratory Medicine, Radboud University Medical Center, Nijmegen, Netherlands; ^2^Department of Biochemistry, Radboud University Medical Center, Nijmegen, Netherlands

**Keywords:** Treg, FOXP3, TNF, anti-TNF, IL-17A, JAK, TCR

## Abstract

Maintenance of regulatory T cells CD4^+^CD25^high^FOXP3^+^ (Treg) stability is vital for proper Treg function and controlling the immune equilibrium. Treg cells are heterogeneous and can reveal plasticity, exemplified by their potential to express IL-17A. TNFα-TNFR2 signaling controls IL-17A expression in conventional T cells via the anti-inflammatory ubiquitin-editing and kinase activity regulating enzyme *TNFAIP3/*A20 (tumor necrosis factor-alpha-induced protein 3). To obtain a molecular understanding of TNFα signaling on IL-17 expression in the human effector (_eff_Treg, CD25^high^CD45RA^−^) Treg subset, we here studied the kinome activity regulation by TNFα signaling. Using FACS-sorted naïve (_naïve_Treg, CD25^high^CD45RA^+^) and _eff_Treg subsets, we demonstrated a reciprocal relationship between TNFα and IL-17A expression; _eff_Treg (TNFα^low^/IL-17A^high^) and _naïve_Treg (TNFα^high^/IL-17A^low^). In _eff_Treg, TNFα-TNFR2 signaling prevented IL-17A expression, whereas inhibition of TNFα signaling by clinically applied anti-TNF antibodies led to increased IL-17A expression. Inhibition of TNFα signaling led to reduced *TNFAIP3* expression, which, by using siRNA inhibition of *TNFAIP3*, appeared causally linked to increased IL-17A expression in _eff_Treg. Kinome activity screening of CD3/CD28-activated _eff_Treg revealed that anti-TNF-mediated neutralization led to increased kinase activity. STRING association analysis revealed that the TNF suppression _eff_Treg kinase activity network was strongly associated with kinases involved in TCR, JAK, MAPK, and PKC pathway signaling. Small-molecule-based inhibition of TCR and JAK pathways prevented the IL-17 expression in _eff_Treg. Together, these findings stress the importance of TNF-TNFR2 in regulating the kinase architecture of antigen-activated _eff_Treg and controlling IL-17 expression of the human Treg. These findings might be relevant for optimizing anti-TNF-based therapy and may aid in preventing Treg plasticity in case of Treg-based cell therapy.

## Highlights

- Naïve and effector CD4^+^ regulatory T cells have a reciprocal IL-17A–TNFα relationship; _eff_Treg (TNF^low^/ IL-17A^high^) and _naïve_Treg (TNF^high^/ IL-17A^low^).
- TNFα-TNF receptor-2 signaling regulates IL-17A expression via ubiquitin-editing *TNFAIP3*/A20 protein in _eff_Treg.
- TNFα suppresses T-cell receptor and Janus kinase protein activity and promotes IL-17A expression in _eff_Treg.
- siRNA-mediated *TNFAIP3* inhibition of _eff_Treg, similar to TNFα signaling inhibition by anti-TNF treatment, leads to enhanced *IL17A* expression.
- TNFα signaling regulates the kinase architecture of antigen-activated _eff_Treg.

## Introduction

Regulatory CD4^+^CD25^high^FOXP3^+^ T cells (Treg) are essential for human immune homeostasis (1). Human Treg cells reveal heterogeneity and contain multiple cell subsets that are characterized by differential expression of maturation, activation, and migration markers (2). At birth, the majority of the Treg are naïve (3), while later in life, the frequencies of CD45RA^−^ memory (effector) Treg increase at the expense of naïve Treg frequencies (4). Naïve (_naïve_Treg) and effector (_eff_Treg) Treg have distinct transcriptional, proteomic, metabolic, as well as enhancer and promoter landscapes (5–7).

Effector Treg cells were shown to express pro-inflammatory cytokines such as the autoimmune associated pro-inflammatory cytokine IL-17A, but also naïve Treg was found to produce IL-17A albeit at lower frequencies (5, 8). IL-17A-producing Treg have been observed in human inflammatory diseases such as psoriasis and IBD, suggesting that they contribute to the inflammatory process as has been demonstrated in mouse models (9–14). Although some cues that regulate IL-17A expression by Treg have been identified, including mTOR inhibition (15), CD28 superagonist stimulation (16), and platelet microparticle interaction (17), our mechanistic understanding of IL-17A expression by Treg is limited, let alone that this information is available for naïve and effector Treg. Recently, it has been elucidated that TNFR2 signaling is vital to establish Treg stability by promoting FOXP3 expression and inhibiting secretion of pro-inflammatory cytokines like IL-17A and IFNγ (18, 19). In conventional CD4+ memory T cells, inhibition of TNFR2 signaling by anti-TNF led to reduced expression of the anti-inflammatory regulator tumor necrosis factor-alpha-induced protein 3 (*TNFAIP3*, also known as A20), and as a consequence, this resulted in increased IL-17A expression (20). *TNFAIP3/*A20 acts as a ubiquitin-editing enzyme that regulates multiple other signaling pathways such as IL-17R (21) signaling and kinase activity [e.g., PKC (22), TCR (23), and MAPK (24)].

TNF-TNFR2 signaling appears essential for human Treg expansion and proper function and additionally an autologous TNFα signaling feedback loop has been proposed that regulates IL-17A expression in human Treg (18, 19, 25–29). Anti-TNF therapy is successfully used for the treatment of severe chronic inflammatory diseases such as inflammatory bowel diseases, psoriasis, psoriatic arthritis, and rheumatoid arthritis (30–33). Paradoxically, it has been observed that in 0.6–5% of the patients treated with anti-TNF medication, this might unintentionally trigger specific forms of immune pathology, suggesting that inhibition of anti-TNF therapy affects Treg function (34–37). If and how naïve and effector Treg are affected by inhibition of TNFα is not known.

We hypothesize that TNFα signaling controls IL-17A expression in Treg by interfering at the level of kinase activity, which we here explored in _eff_Treg. We demonstrate that inhibition of TNFα signaling by anti-TNF *in vitro* led to increased IL-17A expression. Down-regulation of the anti-inflammatory mediator *TNFAIP3* played a role in this process. Comprehensive kinome analysis revealed that inhibition of TNFα signaling in _eff_Treg unexpectedly led to an increase of a kinase activity network containing TCR-linked kinases and immune signaling pathway such as the JAK. Small-molecule-based inhibition of these pathways prevented the anti-TNF-induced IL-17A expression in _eff_Treg.

## Results

### _naïve_Treg and _eff_Treg Cells Reveal a Reciprocal IL-17A—TNFα Relationship

**Figure 1:**
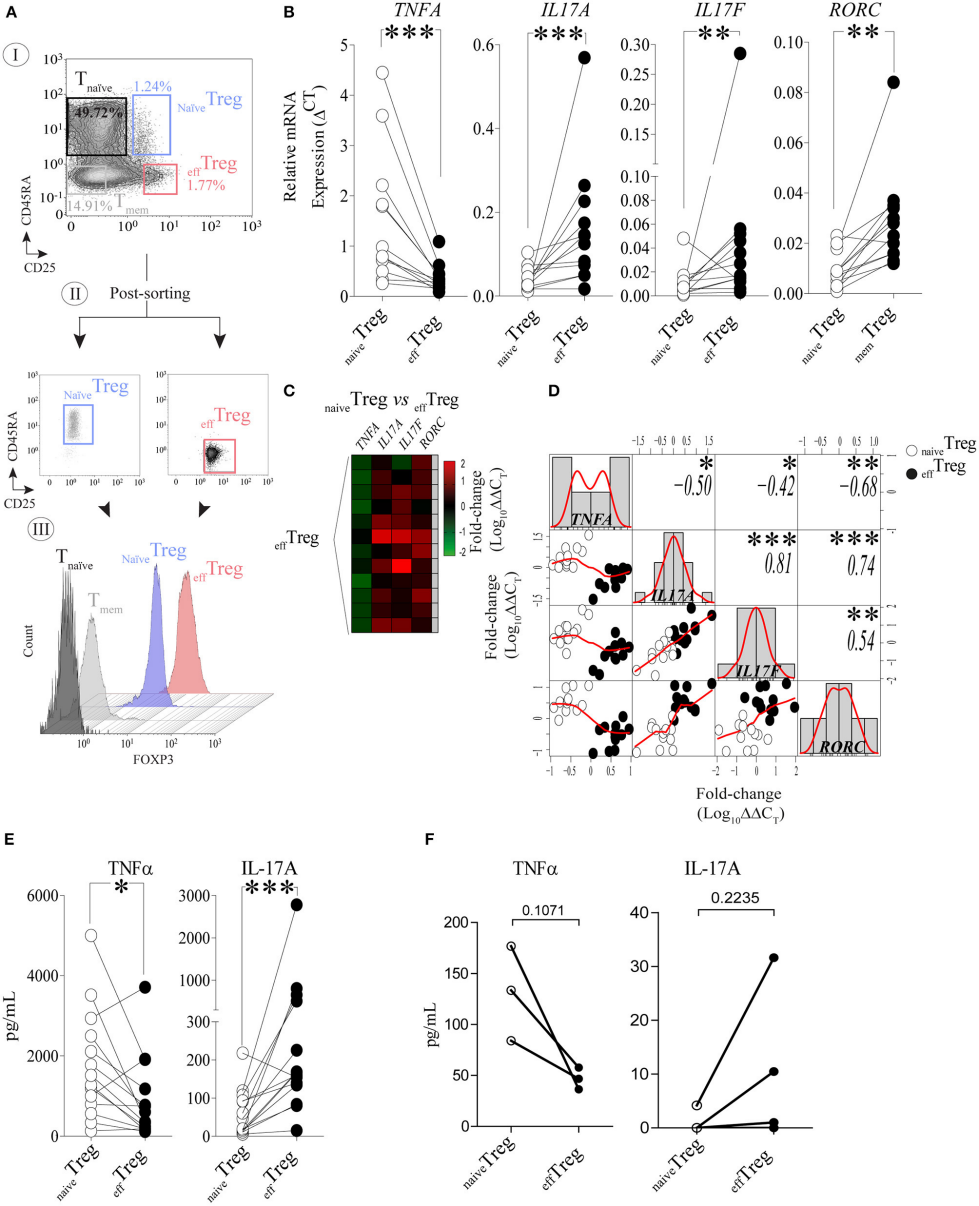
Reciprocal TNFα and IL-17A expression in human _naïve_Treg and _eff_Treg cells. **(A)** An example of the FACS sorting strategy of _naïve_Treg and _eff_Treg based on CD4, CD45RA, and CD25 expression (I. dotplots), post-sorting analysis (II. dotplots) and confirmation of FOXP3 expression in the sorted cell population (III. histograms). Conventional CD4^+^CD45RA^−^CD25^−^ naïve T cells (T_naïve_), and CD4^+^CD45RA^−^CD25^−^ memory T cells (T_mem_) were sorted and displayed for comparison of FOXP3 expression levels (III). **(B)** RT-qPCR gene expression of *TNFA, IL17A, IL17F*, and *RORC* in _naïve_Treg and _eff_Treg after 20 h of PMA and ionomycin stimulation (*n* = 12). **(C)** Heatmap displaying the fold change of transcripts expression in _eff_Treg within different donors (rows). _naïve_Treg were used as reference to calculate the fold change. **(D)** Multiple correlation matrix depicting the correlation of gene expression in both Treg subsets (_naïve_Treg [open dots] and _eff_Treg [closed dots]). Sample distribution (histogram) is shown, linear regression is also plotted (red lines), whereas *p*-value significance and *r-*values are displayed based on Pearson correlation test. *Y* and *X* axes depict the log10-fold change of *TNFA, IL17A, IL17F*, and *RORC* expression. Each column represents a gene; in every intersection (rows), we observe the correlation between genes. **(E)** Presence of the cytokines TNFα and IL-17A in culture supernatant after overnight stimulation of Treg subsets using PMA and ionomycin. Cytokines were measured using Luminex (*n* = 14). **(F)** Presence of TNFα and IL-17A in culture supernatants of αCD3/CD28/rhIL-2 activated Treg subsets after 5 days of culture (*n* = 3, mean ± SEM). For statistical analysis, Wilcoxon matched-pairs signed-ranks test **(B,E)**, or two-way ANOVA followed by a Bonferroni *post-hoc* test **(F)** were used. *p < 0.05, **p < 0.01,***p < 0.001, ns, not significant.

To investigate the link between TNFα and IL-17A expression in naïve and effector Treg, FACS-sorted _naïve_Treg (CD4^+^CD45RA^+^CD25^+^) and _eff_Treg (CD4^+^CD45RA^−^CD25^high^) (Figure 1A) derived from healthy volunteers were stimulated with PMA plus ionomycin, and subsequently *TNFA, IL17A, IL17F*, and *RORC* (RORɤt) expression was accessed by RT-qPCR (Figure 1B). As compared to _eff_Treg, _naïve_Treg expressed significantly lower levels of *IL17A, IL17F, and RORC (p* = 0.0005, *p* = 0.0093, and *p* = 0.0016, respectively), while *TNFA* expression was higher (*p* = 0.0002) (Figure 1B). Next, we compared the fold change in gene expression between the Treg subsets and observed a reciprocal gene expression signature for *TNFA, IL17A, IL17F*, and *RORC* (Figure 1C). Correlation analysis revealed a reciprocal relationship between *TNFA* and *IL17A* (*r* = −0.50)*, IL17F* (*r* = −0.42), and *RORC* (*r* = −0.68) (Figure 1D). As expected, a strong positive correlation between IL17A/IL17F (*r* = 0.81), IL17A/RORC (*r* = 0.74), and IL17A/RORC (*r* = 0.54) was observed. The inverse relationship was also confirmed at the protein level upon PMA plus ionomycin stimulation (Figure 1E) or αCD3/CD28 stimulation of FACS-sorted Treg (Figure 1F). As compared to _eff_Treg, _naïve_Treg hardly produced IL-17A, but showed an increased production of TNFα. Analysis of conventional T cells further supported the uniquely high production of IL-17A in these _eff_Treg, as the numbers of IL-17A/FOXP3-positive cells in FACS-sorted naïve or memory CD4^+^CD25^−^ T cells were very low (Figure S1).

### TNFα-TNF Receptor-2 Signaling Regulates IL-17A Expression via Ubiquitin-Editing *TNFAIP3*/A20 Protein in Effector CD4^+^ Regulatory T Cells

**Figure 2:**
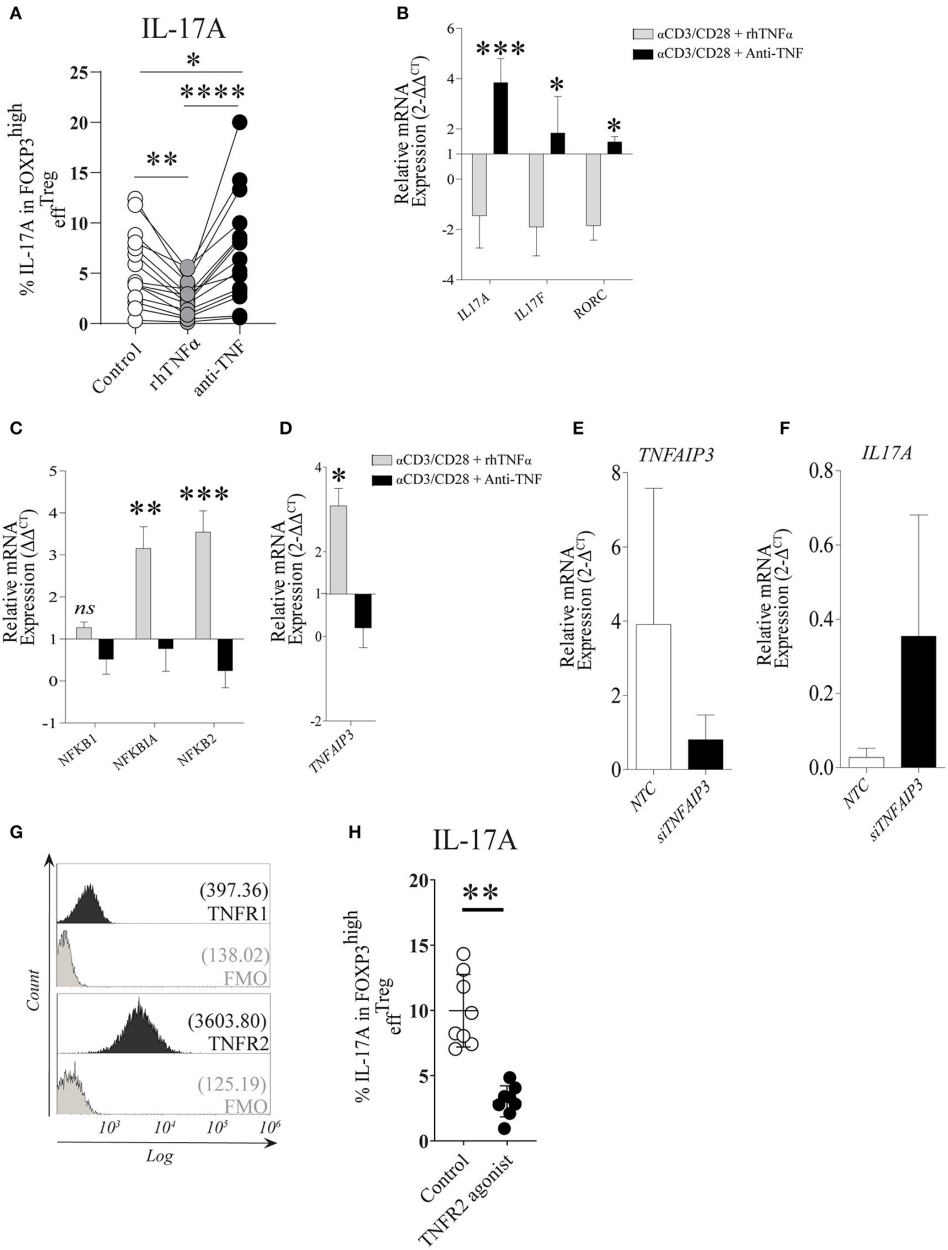
TNFα-TNFR2 signaling reduces IL-17A expression in activated _eff_Treg, conceivably via the anti-inflammatory regulator *TNFAIP3/A20*. **(A)** Flow cytometry of intracellular IL-17A expression in FOXP3^high^_eff_Treg that were stimulated with αCD3/CD28/rhIL-2 for 5 days in the absence or presence of rhTNFα or anti-TNF (*n* = 15). **(B)** RT-qPCR gene expression of *IL-17A, IL-17F*, and *RORC*, **(C)** NFκB target genes *NFKB1, NFKB1A, NFKB2* (*n* = 5), and **(D)** *TNFAIP3* (*n* = 8) at day 4 of culture. **(E,F)** *TNFAIP3* and *IL-17A* gene expression of non-targeting-gene control (NTC) and *siTNFAIP3*_eff_Treg after 6 days under αCD3/CD28/rhIL-2 stimulation (*n* = 3). **(G)** Histogram depicting the expression of TNFR1 and TNFR2 on _eff_Treg directly after FACS sorting (*n* = 9). **(H)** Flow cytometry of IL-17A expression in FOXP3^high^_eff_Treg that were stimulated with αCD3/CD28 beads plus rhIL-2 with or without TNFR2 agonist for 5 days (*n* = 9). All data are shown as mean ± SEM. For statistical analysis, a Friedman test followed by Dunn's multiple comparison test **(A)**, a two-way ANOVA followed by a Bonferroni posttest **(B,C)**, and a Wilcoxon matched-pairs signed-rank test **(D,G,H)** were used. *p < 0.05, **p < 0.01,***p < 0.001, ****p < 0.0001, ns, not significant.

Under the stimulation conditions mentioned above, _eff_Treg, but not _naïve_Treg, demonstrated a clear capacity to produce IL-17A; therefore, we focused our further experiments primarily on _eff_Treg. To analyze if TNFα signaling regulates IL-17A expression in _eff_Treg, FACS-sorted _eff_Treg were stimulated with αCD3/CD28-beads plus rhIL-2 and supplemented with either soluble recombinant human (rh)TNFα or the anti-TNFα agent etanercept (ETN, here referred to as anti-TNF), which is a fusion protein of TNF receptor 2 and IgG1 Fc, which neutralizes TNFα and prevents TNFα signaling. Supplementation of rhTNFα as compared to supplementation of anti-TNF, resulted in a significant reduction of IL-17A expressing FOXP3+ _eff_Treg (*p 3.19e-07*) (Figure 2A). At the transcriptional level, we demonstrated that supplementation of rhTNFα suppressed IL-*17A*, IL-*17F*, and *RORC* gene expression in _eff_Treg (Figure 2B). These data support the idea that TNFα signaling controls IL-17A expression in _eff_Treg.

TNFα binding to its receptors (TNFR1 and TNFR2) leads to a cascade of intracellular events that culminate in NFκB translocation to the nucleus and subsequent transcription of NFκB target genes *NFKBIA* (encode Iκ*βα*), *NFKB1* (encode p50), and *NFKB2* (encode p52) (38, 39). Therefore, we analyzed the effect on the expression of NFκB target genes in _eff_Treg after αCD3/CD28 stimulation with and without supplementation of rhTNFα or anti-TNF. Supplementation with rhTNFα led to a significant increase of *NFKBIA and NFKB2* expression, indicating that TNFα signaling promotes the expression of NFκB target genes, an indication of NFκB activation during Treg activation, while anti-TNF suppressed the NFκB pathway (Figure 2C). We previously found that TNFα signaling enhanced *TNFAIP3* (tumor necrosis factor-induced protein 3) expression in conventional T cells (20). *TNFAIP3* encodes the ubiquitin-editing enzyme A20, which in turn regulates NFκB activity. Here, we also observed that TNFα signaling regulated *TNFAIP3* expression in _eff_Treg (Figure 2D). To demonstrate causality between suppression of *TNFAIP3* and enhanced expression of IL-17A, we carried out a small interfering RNA assay (siRNA) to inhibit *TNFAIP3* transcription. siRNA-mediated *TNFAIP3* inhibition of _eff_Treg, similar to TNFα signaling inhibition by anti-TNF treatment, led to enhanced *IL-17A* gene expression (Figures 2E,F).

As TNFα can bind to both TNFR1 and TNFR2, we measured the expression of these receptors on freshly isolated _eff_Treg and demonstrated that they expressed TNFR2, but TNFR1 was hardly detected (Figure 2G). The latter agrees with previous studies (20, 40) and suggests that TNFα-mediated regulation of IL-17A expression in _eff_Treg might be primarily mediated via the TNFR2. To examine this, αCD3/CD28-stimulated _eff_Treg were cultured in the absence and presence of a specific TNFR2 agonist for 5 days. TNFR2 agonist stimulation led to a reduction in the percentages of IL-17A expressing FOXP3+ cells (Figure 2H). This indicates that IL-17A expression in _eff_Treg subsets is regulated via TNFα-TNFR2 signaling. Together, these data suggest that TNFα signaling via TNFR2 promotes the expression of the anti-inflammatory mediator *TNFAIP3/*A20, which seems to prevent IL-17A expression in regulatory T cells, as ablation of TNFα signaling suppresses *TNFAIP3/*A20 and results in increased IL-17A expression in human Treg.

### TNFα Suppresses T-Cell Receptor and Janus Kinase Protein Activity and Regulates IL-17A Expression in Effector Regulatory T Cells

**Figure 3:**
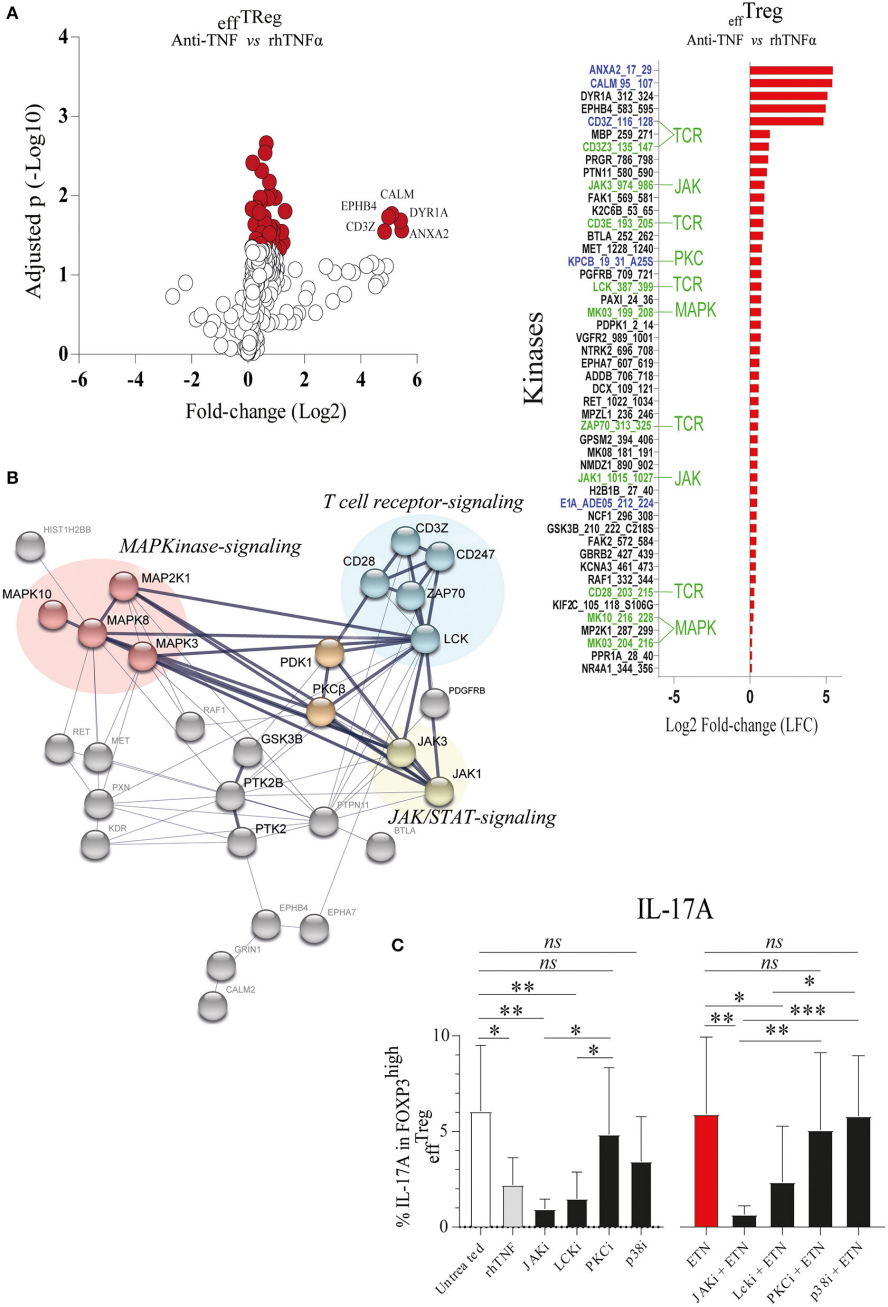
TNFα signaling in _eff_Treg suppresses TCR and JAK kinase activity, leading to regulation of IL-17A expression. _eff_Treg were stimulated with αCD3/CD28 beads and rh-IL-2 in the presence of rhTNFα or anti-TNF. On day 4, phosphoserine/threonine kinase (STK) and phosphotyrosine kinase (PTK) activity of cells were Analyzed using a kinome activity array. **(A)** Left panel: Volcano plot showing the fold change in kinase activity and adjusted *p*-values (red symbols, *p* < 0.05; *n* = 4) in STK and PTK kinase activity. Right panel: Fold change in the kinases identified by comparing anti-TNF with rhTNFα conditions. Of note, TNFα was used as reference to calculate the fold change. Green texts indicate unique kinases that show increased activity upon comparison of anti-TNF to rhTNFα conditions; Blue texts represent kinases with enhanced activity upon comparison of comparing anti-TNF to the control (αCD3/CD28 stimulated without rhTNF or anti-TNF). **(B)** Cumulative STRING^©^ protein network analysis based on the identified kinases listed in **(A)**. **(C)** Flow cytometry of intracellular IL-17A expression in FOXP3^high^_eff_Treg. Pathway inhibition validation assays applying small chemical molecules in the stimulation assay as described above (mean ± SEM, *n* = 7). JAKi, JAK inhibitor (tofacitinib); Lcki, Lck inhibitor (A420983); PKCi, PKC inhibitor (AEB071); and p38i, p38MAPK inhibitor (UR13870). ANOVA Dunnett's testing **(A)** and Friedman test followed by Dunn's multiple comparisons test **(C)** were used. *p < 0.05, **p < 0.001, ***p 0.0001, ns, not significant.

*TNFAIP3*/A20 has been demonstrated to regulate critical proteins involved in TCR (23), TNFα (41), IL-17R (21), and Wnt signaling (20, 42). Recently, we demonstrated that the prevention of TNFα signaling in conventional CD4^+^ memory T cells leads to inhibition of *TNFAIP3*/A20 expression, which subsequently leads to enhanced IL-17A expression (20). *TNFAIP3*/A20 has been shown to regulate kinase activity (21, 23). To better understand kinase regulation by TNFα signaling in _eff_Treg, we here profiled the activity of ~300 kinases in FACS-sorted _eff_Treg following stimulation with αCD3/CD28 beads in the absence or presence of anti-TNF or rhTNFα. Subsequently, we analyzed the threonine/serine and tyrosine kinase activity using a multiplex human kinase activity array. This kinome array employs ~300 peptide substrates with known phosphorylation sites and provides a reliable and high-throughput kinase profiling tool for further pathway elucidation (see *Materials and Methods*) (43). We found 30 unique and differentially activated kinases following anti-TNF vs. rhTNFα supplementation comparison (Figure S2). For the kinase activity profiling, we focused on the two most extreme states of TNF pathway signaling and addressed the differential kinase activity profile following _eff_Treg activation following TNF vs. anti-TNF supplementation. The obtained kinome data were visualized using a volcano plot that shows the fold change of kinase activity and the associated level of significance (*p*-values) (Figure 3A, left panel; raw data Table S1). We found that inhibition of TNFα signaling, as compared to the supplementation of rhTNFα, in activated _eff_Treg significantly promoted the activity of multiple kinases (red symbols indicate *p* < 0.05). The ranked log2-fold changes of kinase activity are shown in the right panel of Figure 3A. Notably, several of the kinases were related to TCR signaling [CD3ζ (CD247), CD3ε, ZAP70, and Lck] (44). Also, cell cycle regulating (CALM, CD28, GSK3B, MAPK3, PGR, and JAK3) (45, 46) and apoptosis (ANXA2, Annexin V) (47)-related kinases were induced.

To obtain a more comprehensive understanding of the kinase network and cellular pathways regulated by neutralization of TNFα, the kinases that were significantly activated following anti-TNF mAb treatment were analyzed using STRING (*Search Tool for the Retrieval of Interacting Genes/Proteins*). STRING is a web-based biological resource (https://string-db.org) of known and predicted protein–protein interactions enabling prediction of the functional protein association network of a group of given proteins by estimating the likelihood of meaningful biological interactions (48). In our analysis, we used the highest confidence interaction score (0.900) to associate all kinases that were significantly activated following anti-TNF treatment as listed in the right panel of Figure 3A. STRING association analysis demonstrated that inhibition of TNFα signaling in activated _eff_Treg involved prominent immune signaling pathways such as the PKC, p38-MAPK, and JAK pathways, which were all linked to TCR signaling [CD3ζ (CD247) and CD3ε] (Figure 3B). Previously, these pathways were shown to be associated with the induction of IL-17A expression (49–52).

To validate if the predicted pathways were indeed involved in rhTNFα-induced suppression of IL-17A expression in _eff_Treg, FACS-sorted _eff_Treg were activated in the presence or absence of anti-TNF and specific kinase inhibitors of JAK/STAT (Tofacitinib), PKC (AEB071, Sotrastaurin), or p38 MAPK (UR13870). For the inhibition of TCR signaling, an Lck inhibitor (A420983) was applied. We demonstrated that suppression of JAK, and Lck kinases, but not PKC and p38, prevented the expression of IL-17A expression in _eff_Treg that were activated under TNFα signaling inhibiting or not (Figure 3C). In fact, suppression of JAK and Lck inhibited the expression of IL-17 similar to the TNF supplementation condition. The inhibitors tested did not affect FOXP3 expression (Figure S3).

**Figure 4:**
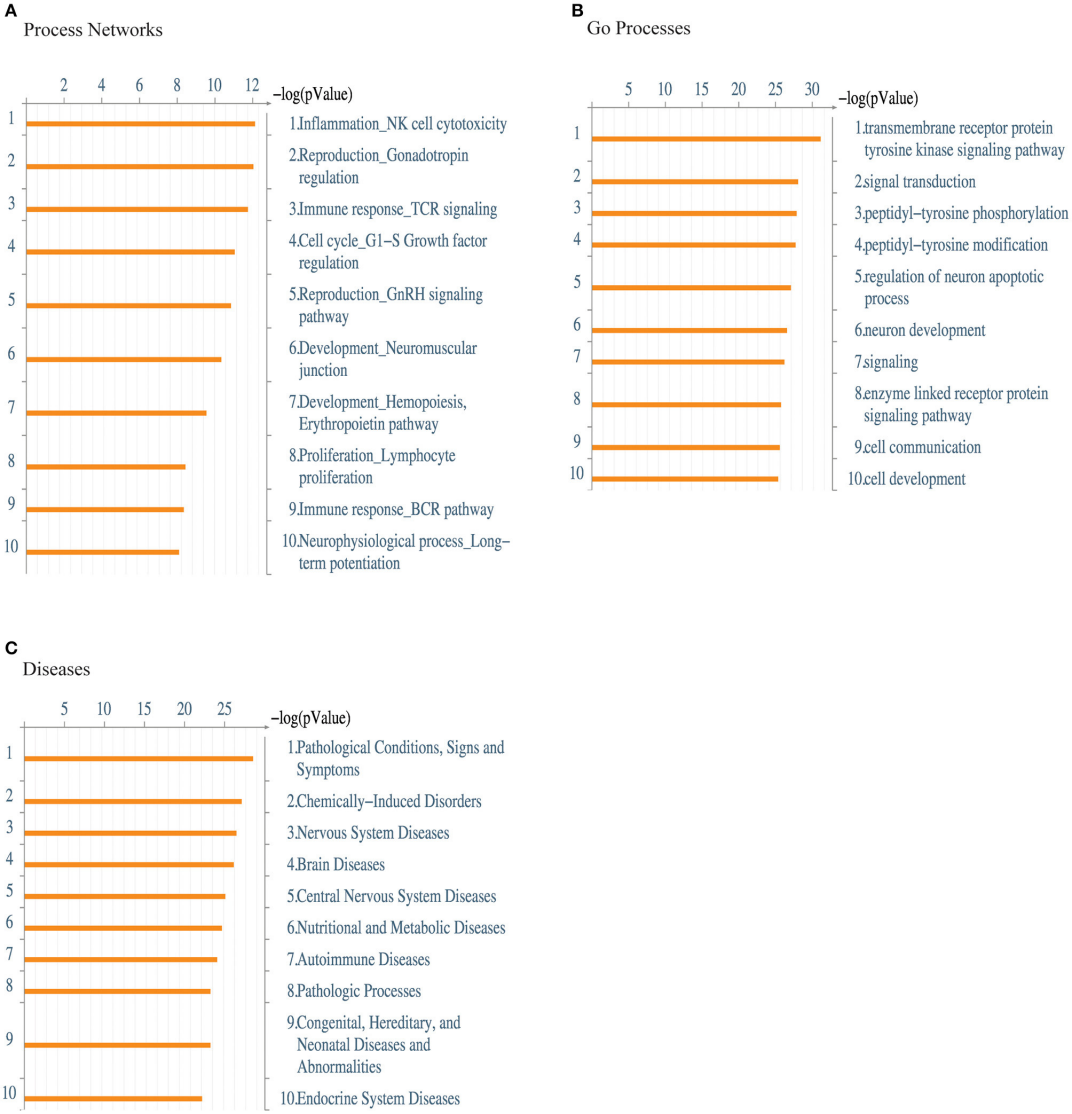
Enrichment analysis of the kinome array data. Functional ontology enrichment analysis using the MetaCore™ database reveals **(A)** the distinct biological networks, **(B)** the different biological processes, and **(C)** various diseases related to kinases identified in Figure 3. The probability of a random intersection between the set of kinases with ontology entities was estimated with the “*p*” value of the hypergeometric intersection. A lower “*p*” value means higher relevance of the entity to the dataset, which appears in higher rating for the entity.

Next, we performed a functional ontology enrichment analysis of the most significant biological process networks, processes, and diseases by submitting the kinase data that we identified in activated _eff_Treg following supplementation vs. inhibition of TNFα to MetaCore™ database analysis. Significant enriched MetaCore™ GO process networks involved immune response-TCR signaling, cell cycle regulation, and lymphocyte proliferation (Figure 4A). The most significantly enriched MetaCore™ Go processes based on the submitted kinases were kinase signaling pathways via transmembrane receptor protein tyrosine, signal transduction processes, and tyrosine phosphorylation and modification (Figure 4B). Furthermore, there was an enrichment of cell communication and cell development processes. MetaCore™ Go diseases indicated a strong enrichment of autoimmune disease, next to other pathological conditions ranging from the nervous system, nutritional, and metabolic disorders (Figure 4C). Together, these data demonstrated that CD3 and CD28 activation of _eff_Treg in the absence of TNF-signaling by anti-TNF treatment promotes tyrosine kinase activity of relevant TCR-associated signaling pathways.

## Discussion

Human Treg can express the pro-inflammatory cytokine IL-17A under specific conditions; a phenomenon referred to as Treg plasticity (5, 8). The molecular mechanisms regulating this phenomenon are not well-understood. In our current work, we demonstrate that TNFα signaling regulates IL-17A expression in _eff_Treg by controlling a kinase activity network that includes TCR linked kinases and other prominent immune signaling kinase pathways such as the JAK pathway. Also, TNFα-mediated regulation of the anti-inflammatory mediator *TNFAIP3*/A20 appeared crucial to control IL-17A expression by _eff_Treg. TNFR2 is the main receptor for TNFα signaling in Treg. TNFR2 stimulation has been demonstrated to support Treg stability (18, 19, 25, 53), whereas the effect of TNF signaling on the stability of Treg is ambiguous (54, 55). Here, we show that TNFR2 is highly expressed on human _eff_Treg, and TNF-TNFR2 signaling in _eff_Treg acts as a negative regulator of IL-17A expression by controlling TCR and JAK signaling.

STRING association analysis revealed that inhibition of TNFα signaling is associated with increased TCR associated signaling of CD3ζ, CD3ε, ZAP70, and Lck, indicating that TNFα signaling in _eff_Treg functions as a rheostat of TCR signal transmission. Although information of TNFα stimulation on the TCR signaling in Treg is lacking, it has been shown in CD4+ T cells of both mice and man that TNFα stimulation results in specific down-regulation of TCRζ expression and impaired TCR/CD3 signaling, including phosphorylation of the TCRζ, CD3ε, ZAP-70 tyrosine kinase, and linker for activation of T cells (LAT) (56). TCR signaling is essential for both effector and regulatory T cells (57). Treg have a more extensive TCR repertoire than effector T cells, and TCR signaling is crucial for proper Treg function (58–61). Signaling via the T cell antigen receptor of Treg is critical for FOXP3 expression and their suppressive activity. Mutations resulting in signaling-deficient TCRζ chains led to increased Treg numbers with higher suppressive activity (62–64). Reduced TCR signaling will alleviate downstream signaling and favor Treg cell lineage commitment. TNFα signaling, as we demonstrate here, seems to safeguard TCR-related kinase activity in _eff_Treg and stabilize Treg function as illustrated by preventing IL-17A expression. Note that anti-TNF had a mild effect on the induction of IL-17A expression in _eff_Treg, which is in contrast to its clear induction of IL-17A in conventional memory T cells (20). This phenomenon may be caused by the poor intrinsic capacity of _eff_Treg to produce TNFα *in vitro*. In fact, highly pure FACS-sorted _eff_Treg barely produced TNFα (41.35 pg/ml ± 6.75), whereas memory conventional T cells produced significantly higher levels (335.7 pg/ml ± 65.33, *n* = 4) (data not shown).

Next to TCR-derived signals, Treg integrates inputs from cytokine, chemotactic, and metabolic cues to fulfill their function optimally. Proximal cytokine signaling often takes place via JAK-STAT signaling (65). *IL-17A* gene transcription is associated with JAK-STAT3 signaling (66). Inhibition of TNFα signaling using anti-TNF inhibitor ETN was associated with increased JAK1 and JAK3 kinase activity in αCD3/CD28 stimulated _eff_Treg. Inhibition of JAK1 and JAK3 kinase activity by the clinically applied JAK inhibitor tofacitinib prevented IL-17A expression in anti-TNF-treated _eff_Treg, suggesting that TNFα signaling is involved in driving JAK/STAT signaling. Although TNFα is not a prototypic JAK/STAT activating cytokine, the anti-inflammatory molecule A20 (encoded by *TNFAIP3*) that is a downstream target of TNFα signaling acts as a regulator of STAT (67, 68). The absence of A20 in myeloid cells resulted in enhanced STAT1-dependent inflammation (68). This relationship needs to be confirmed in _eff_Treg.

Although anti-TNF therapy is improving the life quality of many patients with chronic inflammatory diseases, 10–20% of patients do not respond to the treatment while 0.6–5% of patients treated with TNF inhibitors reveal paradoxical immune-mediated inflammatory side effects (36, 37). Although the mechanism of the latter phenomenon is not fully understood, it might be of interest to consider an additional JAK inhibitor treatment such as tofacitinib or other JAK inhibitors to prevent the putative IL-17A expression by Treg. Also, regarding Treg-based immune therapy in transplantation or autoimmunity, the clinical design has started to consider strategies to minimize the risks of Treg plasticity (69) at the time of ex vivo production and following *in vivo* administration (70, 71). Our results suggests that TNFα-TNFR2 signaling or inhibition of JAK signaling might favor Treg stability. Along with this line of reasoning, it has been demonstrated that JAK inhibition (72) as well as TNFR2 stimulation (18, 19) support human Treg function and prevent Treg plasticity.

In conclusion, we demonstrated an inverse production of TNFα and IL-17A between human naïve and effector Treg cells. Supplementation of rhTNFα led to a down-regulation in the frequency of IL-17A-producing _eff_Treg, mainly via the activation of NFkB pathway as well as the up-regulation of *TNFAIP3/A20* expression. TNFR2 receptor seems to play a crucial role since we hardly detected any expression of TNFR1 on _eff_Treg and treatment of _eff_Treg with TNFR2 specific agonist resulted in a similar inhibition of IL-17A production. Accordingly, inhibition of TNFα signaling using the clinically applied anti-TNF inhibitor ETN led to decreased *TNFAIP3* and increased *IL-17A* expression, a phenomenon similar to what is observed in human conventional memory CD4+ T cells. Kinome activity screening of αCD3/CD28 stimulated _eff_Treg revealed that anti-TNF led to an increase in kinase activity of multiple kinases including CD3ζ (CD247) and LcK. A functional ontology enrichment analysis indicated that these kinases were highly associated with different immune response signaling pathways including TCR-, JAK-mediated pathways. We propose that these findings might be relevant for optimizing anti-TNF-based therapy and may aid in preventing Treg plasticity in case of Treg-based cell therapy.

## Materials and Methods

### Study Approval

The protocols of this study were performed in agreement with the Declaration of Helsinki and in accordance with the Radboud university medical center (Radboudumc) in Nijmegen, the Netherlands.

### Subjects

Blood buffy coats from voluntary donors were purchased from the Sanquin Blood Bank, Nijmegen, the Netherlands. The volunteers gave written informed consent.

### Regulatory T Cell Isolation

CD4^+^ T cells were isolated using RosetteSep™ Human CD4^+^ T cell enrichment cocktail 25–50 μl of cocktail/ml of blood (StemCell Technologies, Vancouver, Canada) according to the instructions of the supplier. To sort CD4^+^CD25^+^CD45RA^+^ (_naïve_Treg) and CD4^+^CD25^high^CD45RA^−^ (_eff_Treg), the purified CD4^+^ cells were washed and stained with anti-CD25-BV510 (*M-A251*, BD, New Jersey, USA), anti-CD45RA^−^ PE (*4KB5*, Dako, Brüsseler Straße, Germany), CD4-PE-Cy5.5 (*13B8.2*, Beckman-Coulter, California, United States), and FACS-sorted on a FACSAria™ III machine (BD Biosciences, New Jersey, United States). The gating strategy during FACS sorting, post-sorting purity analysis, and confirmation of FOXP3 expression in freshly sorted cell subsets are described in Figure 1A. The purity of the sorted cell populations was 95.3 ± 4.1% (mean ± SD).

### Cell Culture

RPMI-1640 Dutch modified (Gibco, Massachusetts, United States) culture medium, containing sodium bicarbonate and 20 mM HEPES, supplemented with penicillin/streptomycin (100 U/ml), sodium pyruvate (1 mM), glutamine/glutamax, and 10% human pooled serum (HPS, Radboudumc), was used in all experiments. After cell isolation, 2.5 × 10^4^ cells/well were cultured in 96-well U-bottom plates and stimulated with Dynabeads^®^ Human T-Activator CD3/CD28 (αCD3/CD28 beads, 1:5 of bead:cell ratio) (Gibco, Massachusetts, United States) in the presence of recombinant human (rh) IL-2 (rhIL-2, 100 U/ml) (Proleukin Prometheus Laboratories, California, United States). In some conditions, cultures were supplemented with rhTNFα (50 ng/ml, R&D, Minnesota, United States), or TNFα inhibitors etanercept (5 μg/ml; ETN—Enbrel, Pfizer, New York, United States), or TNFR2 agonist (2.5 μg/ml, Clone *MR2-1*, Hycult Biotech, Uden, the Netherlands). To examine the effect of a pharmaceutical inhibitor, tofacitinib (0.112 μM, Pfizer, New York, United States), PKC inhibitor Sotrastaurin (1 μM), Lck inhibitor A420983 (1 μM), or p38α/β kinase inhibitor UR13870 (10 μM) was pre-incubated with the FACS-sorted cells for 30 min before the addition of any stimulus. In some cases, cells were stimulated with PMA (12.5 ng/ml) and ionomycin (500 ng/ml) for 20 h.

### Flow Cytometry

Flow cytometry was performed using a 10-color Navios Flow cytometer (Beckman Coulter, California, United States), which is equipped with blue (488 nm), red (638 nm), and violet (405 nm) lasers. For surface staining, the following antibodies were used: anti-CD3-ECD (*UCHT1*), anti-CD45RA-ECD (*2H4LDH11LDB9*), anti-CD45-KO (*J33*), anti-CD4-PE-Cy5.5 (*13B8.2)*, and anti-CD8-APC-AF700 (*B9.11*) (all from Beckman-Coulter); anti-TNFR1-AF488 (*16803*, R&D); and anti-TNFR2-APC (*22235*, R&D). For intracellular staining, the following antibodies were used: anti-IFNγ-PE-Cy7 (*4S.B3*) and anti-IL-17A-AF-660 (*eBio64DEC17*) (eBioscience, California, United States). Unstained (Fluorescence Minus One, FMO) samples were also measured to help set the gates during data analysis. To evaluate cytokine production, we challenged the cultured Treg subsets for another 4 h with PMA (12.5 ng/ml), ionomycin (500 ng/ml), and Brefeldin A (5 μg/ml) (Sigma-Aldrich, Missouri, United States) before performing the FACS staining process. Briefly, cells were stained with the fixable viability dye-eFluo 780 (FVD, eBioscience) for 30 min at 4°C, following with surface mAb staining, cell fixation, and permeabilization by using the Intracellular Fixation & Permeabilization Buffer Set (eBioscience) and intracellular mAb staining. For flow cytometry data analysis, Kaluza1.5 software (Beckman Coulter) was used.

### Small Interfering RNA Transfection

For small interfering RNA (siRNA) knockdown of *TNFAIP3*, Accell SMARTpool siRNA (Dharmacon, Colorado, United States) was used according to the manufacturer's instructions. Briefly, 1 × 10^5^_eff_Treg cells per well were stimulated with αCD3/CD28 beads (1:5 of bead:cell ratio) in Accell Delivery Medium (Dharmacon) supplemented with rhIL-2 (100 U/ml) and incubated with 1 mmol cyclophilin B siRNA (positive control), or 1 mmol non-targeting control siRNA, or 1 mmol *TNFAIP3* siRNA for 120 h (for siRNA sequences, see Table S2). Quantitative real-time PCR (RT-qPCR) was performed to confirm the knockdown of the target gene expression.

### RT-qPCR

Total RNA was extracted by using the RNeasy Plus Micro Kit (Qiagen) followed by cDNA synthesis using the SuperScript III First-Strand Synthesis System and Oligo(dT)20 primer (Thermo Fisher Scientific, Massachusetts, United States). TaqMan gene expression assays were purchased from Thermo Fisher Scientific (Table S3). RT-PCR was acquired in a 7500 Real-Time PCR System (Applied Biosystems). RT-qPCR cycle values (C_T_) obtained for specific mRNA expression in each sample were normalized to the C_T_ values of human *HPRT1* (endogenous control), resulting in ΔC_T_ values (log ratio of the gene concentrations) that were used to calculate the relative gene expression.

$$\begin{array}{l} \Delta {\text{C}}_{\text{T}=}Mean\;CT-Housekeeping\;gene\;Mean\;CT \end{array}$$

Then, we performed an exponential conversion of ΔC_T_, namely, 2^−ΔCT^ using the following formula:

$$\begin{array}{l} 2^{\wedge }\left(exponential\right)-\;\Delta CT \end{array}$$

2^−ΔCT^ representing the relative gene expression was used in Figures 1B,E,F.

_eff_Treg stimulated in the absence of anti-TNF or rhTNFα were used as a baseline to calculate the relative gene expression in fold change (ΔΔC_T_) for _eff_Treg stimulated in the presence of rhTNFα *vs*. ETN treatment.

$$\begin{array}{l} \Delta \Delta C_{\text{T}}=Mean\;\Delta CT-Mean\;\Delta CT\;reference\;sample\;\left(control\right) \end{array}$$

Subsequently, we performed an exponential conversion of *ΔΔC*_T_, namely, 2^−ΔΔCT^ using the following formula:

$$\begin{array}{l} 2^{-\text{Δ}\Delta \text{CT}}=2^{\wedge }\left(exponential\right)-\;\Delta \Delta CT \end{array}$$

2^−ΔΔCT^ representing the relative gene expression in fold change was employed for Figures 2B–D. In Figures 1C,D, log10 ΔΔC_T_ was employed. The Relative Quantification app (Thermo Fisher Scientific cloud) was used for data analysis.

### Measurement of Cytokines Secretion

The cell culture supernatants were analyzed for the presence of IL-17A, IFNγ, and TNFα using Bio-Plex Pro Human Th17 Cytokine Assays (Bio-Rad, California, United States) according to the manufacturer's instruction. The cytokine concentrations were measured using a Luminex^100^ machine (Luminex Corp., Texas, United States). The lowest limit of detection was <1.870 pg/ml for IL-17A, <2.411 pg/ml for IFNγ, and <2.231 pg/ml for TNFα.

### Protein Kinase Chip Assay

After sorting and stimulations of cells, samples were frozen for further analysis. The protein isolation was performed according to the manufacturer's instruction (P1160, PamGene International B.V., 's-Hertogenbosch, the Netherlands). Kinase activity was measured with PamGene's Protein Tyrosine Kinase (PTK) PamChip (Cat. number 86402) and Serine Threonine kinase (STK) PamChip (Cat. number 87102). Each PTK PamChip array contains 196 peptides immobilized on a porous membrane, whereas each STK PamChip array contains 144 peptides (see the full list of peptides at www.pamgene.com). The peptide sequences (13 amino acids long) harbor phosphorylation sites, defined based on literature or derived from computational predictions and are correlated with one or multiple upstream kinases. A fluorescently labeled anti-phospho-Tyr antibody (PY20) is used to detect the phosphorylation activity of tyrosine kinases present in the sample. For the STK assay, an antibody mix is used to detect the phosphorylated Ser/Thr, and the 2nd FITC-conjugated antibody is used in a detection mix to quantify the phosphorylation signal. BioNavigator software 6.3 (PamGene) was used to determine signal intensities, peptide quality control (QC) and preselection (phosphorylation kinetics, or increase in signal over time, in 25% of the arrays analyzed), Log 2 transformation, ANOVA-Dunnett's testing, and data visualization. Mapping and pathway elucidation analysis were performed using METACORE™ (Clarivate Analytics, PA, USA) and STRING (73). As described by the GeneGo manufacturer's report, the analysis consists in matching the protein IDs of possible targets for the “common,” “similar,” and “unique” sets with protein IDs in functional ontologies in MetaCore (73). The lower *p*-value means a higher relevance of the entity to the dataset, which shows a higher rating for the entity.

### Statistics

Statistical analysis was performed using GraphPad Prism 5.0 for Windows (GraphPad Software, San Diego, California, USA) and R. For experiments with more than two groups of matched samples, we used non-parametric Friedman test followed by Dunn's Multiple Comparison Test, whereas for experiments with only two groups of matched samples, we employed non-parametric Wilcoxon matched-pairs signed-rank test.

## Data Availability Statement

The datasets generated for this study are available on request to the corresponding author.

## Ethics Statement

The studies involving human participants were reviewed and approved by the Radboud university medical center (Radboudumc) in Nijmegen, the Netherlands. The patients/participants provided their written informed consent to participate in this study.

## Author Contributions

PU, IJ, and HK designed the research. PU, OF, XH, HT, and BH performed the experiments. PU, OF, IJ, BH, XH, RS, and HK analyzed the data. PU, XH, IJ, and HK prepared and wrote the final manuscript. All the authors reviewed the paper.

### Conflict of Interest

The authors declare that the research was conducted in the absence of any commercial or financial relationships that could be construed as a potential conflict of interest.

## Supplementary Material

Figure S1 Expression of IL-17A in Conventional and regulatory T cells. Sorted naïve T cells (CD4^+^CD45RA^+^CD25^−^), memory T cells (CD4+CD45RA^−^CD25^−^), naïve Treg (CD4^+^CD45RA^+^CD25^low^) and effector Treg (CD4+CD45RA^−^CD25^high^) were stimulated with anti-CD3/CD28 bead plus rhIL-2. Cells were harvested on day 5 and intracellular FOXP3 and IL-17A expression were detected by FACS staining.

Figure S2 Kinome data analysis. Kinase activity of _eff_Treg following αCD3/CD28 bead plus rhIL-2 activation in the absence or presence of anti-TNF or rhTNFα. Significant changes of kinase activities are presented in a Venn diagram (**A**, left panel) and a bar plot that ranked based on the log2 fold-change of kinase activities. **(B,C)** Bar graphs showing significant changes of kinase activity between anti-TNF and αCD3/CD28 control **(B)** or rhTNF and αCD3/CD28 control **(C)**.

Figure S3 The Janus kinase, Lck, PKC and p38 MAPK inhibitors do not affect FOXP3 expression in _eff_Treg. _eff_Treg were stimulated with αCD3/CD28 beads in the presence or absence of rhTNFα or anti-TNF or small chemical molecules such as JAK inhibitor (tofacitinib), Lck inhibitor (A420983), PKC inhibitor (AEB071) and p38MAPK inhibitor (UR13870) for 5 days. Flow cytometry analysis of intracellular FOXP3 expression (*n* = 5). Data are shown as mean ± SEM.

Table S1 Kinome Log 2-transformed dataset.

Table S2 Target genes used for siRNA interference.

Table S3 Primers used for RT-qPCR.
