# Supplementary material for: TNFα-Signaling Modulates the Kinase Activity of Human Effector Treg and Regulates IL-17A Expression
Source: Front Immunol. 2020 Jan 21;10:3047. doi: 10.3389/fimmu.2019.03047 (PMC6986271; doi:10.3389/fimmu.2019.03047)
Supplement: Figure S1 — Expression of IL-17A in Conventional and regulatory T cells. Sorted naïve T cells (CD4+CD45RA+CD25−), memory T cells (CD4+CD45RA−CD25−), naïve Treg (CD4+CD45RA+CD25low) and effector Treg (CD4+CD45RA−CD25high) were stimulated with anti-CD3/CD28 bead plus rhIL-2. Cells were harvested on day 5 and intracellular FOXP3 and IL-17A expression were detected by FACS staining. [file Data_Sheet_1.PDF]

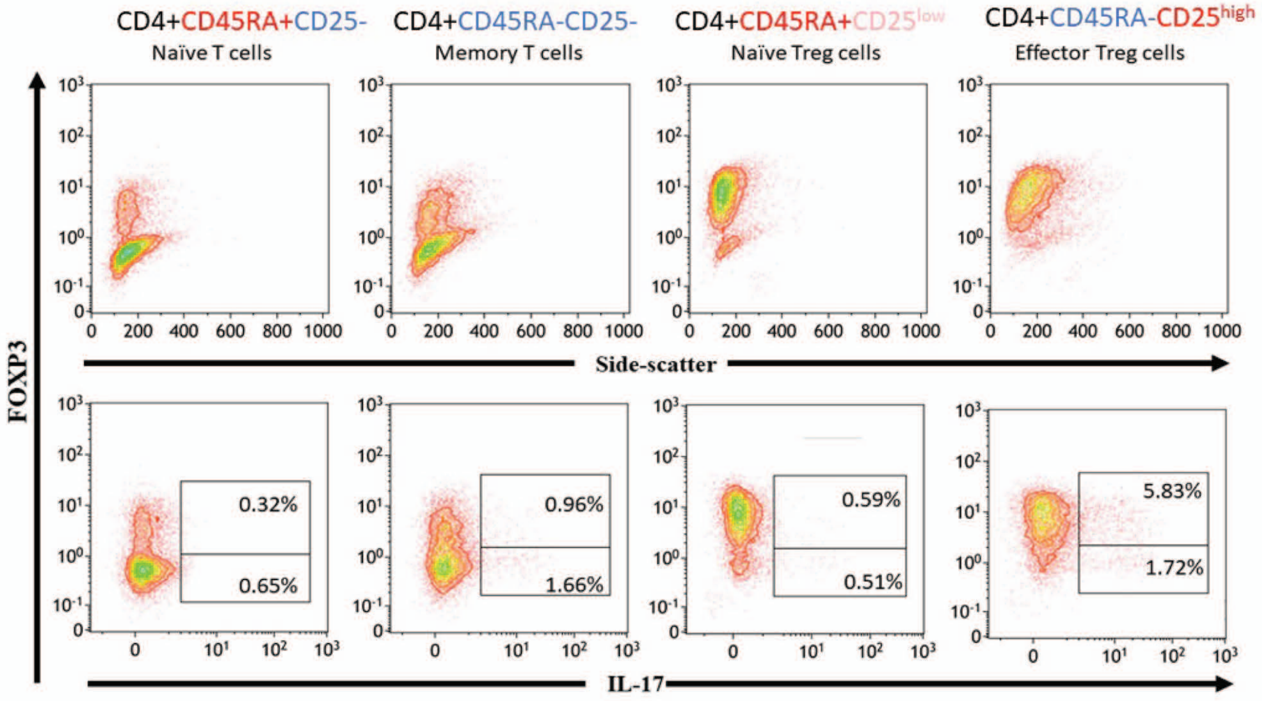

Figure S1. Expression of IL-17A in conventional and regulatory T cells. Sorted naïve T cells (CD4+CD45RA+CD25-), memory T cells (CD4+CD45RA-CD25-), naïve Treg (CD4+CD45RA+CD25low) and effector Treg (CD4+CD45RA-CD25high) were cultured with anti-CD3/CD28 bead activation plus IL-2. On day 5 cells were harvested and intracellular FOXP3 and IL-17A expression were detected by flow cytometry as depicted in the FACS dot plot.

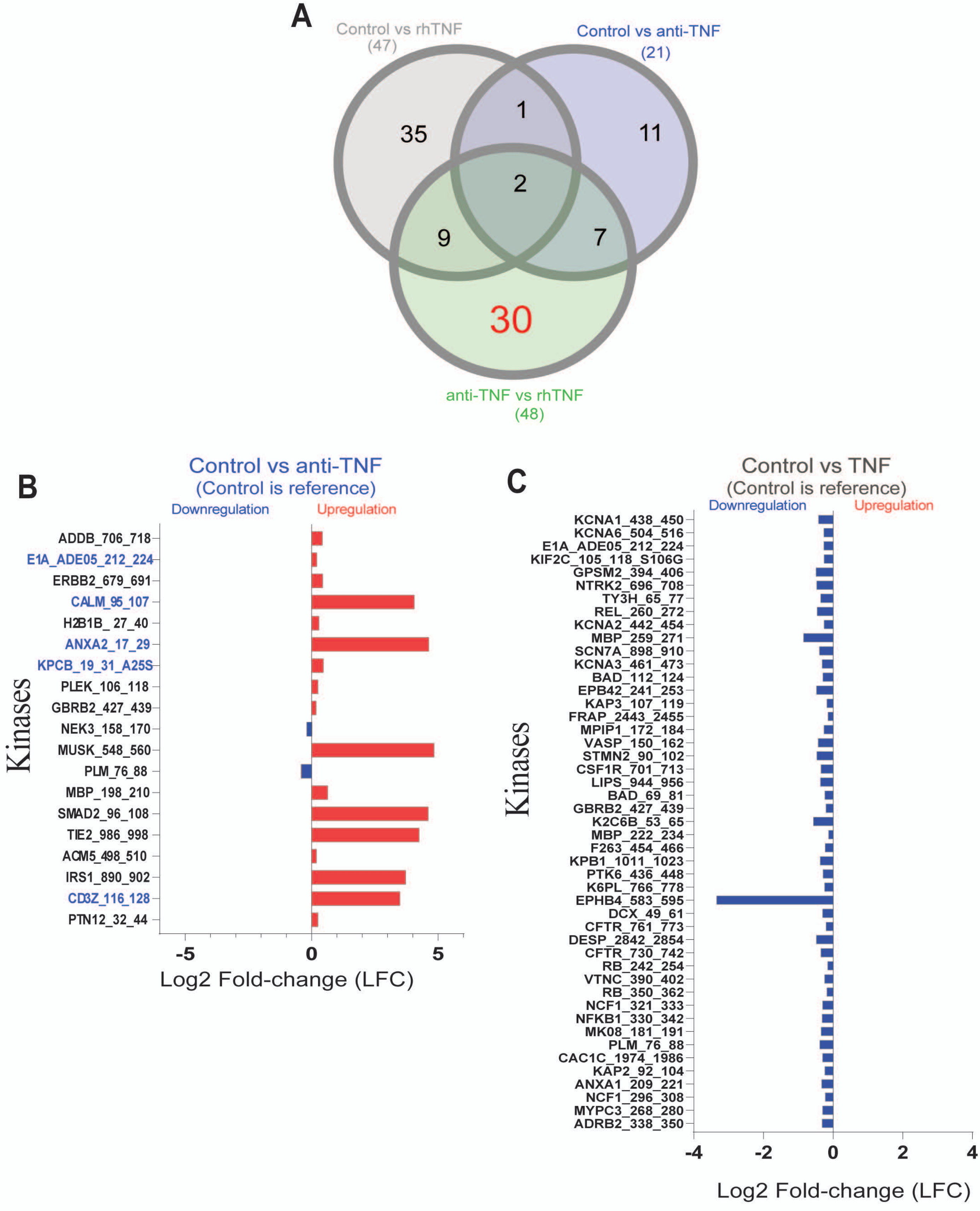

Figure S2. Kinome data analysis. Kinase activity of ${}_{eff}$Treg following anti-CD3/CD28 bead activation plus IL-2 in the absence or presence of anti-TNF (ETN) or rhTNF. Significant changes in kinase activity are presented in the **A.** Venn diagram and were subsequently ranked and plotted based on log2 fold-change. **B.** and **C.** Bar graphs showing only significant different activated kinases and their LOG2 fold-change between untreated condition (Control) and anti-TNF therapy and control vs rhTNF supplementation, respectively.

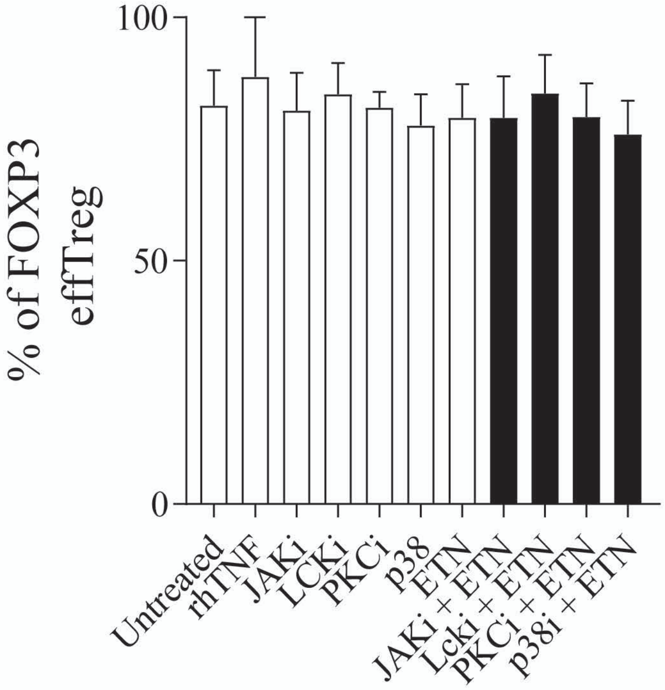

Figure S3. The Janus kinase, Lck, PKC and p38 MAPK inhibitors do not affect FOXP3 expression in $_{eff}$Treg upon activation and supplementation of TNF or TNFα inhibition. $_{eff}$Treg stimulated by αCD3/CD28 beads were supplemented or not with rhTNF or anti-TNF or small chemical molecules such as JAK inhibitor (tofacitinib), Lck inhibitor (A420983), PKC inhibitor (AEB071) and p38MAPK inhibitor (UR13870) for 5 days. Flow cytometry analysis of intracellular FOXP3 expression (*n=5*). Data are shown as mean ± SEM.
