# Supplementary material for: TNFα-Signaling Modulates the Kinase Activity of Human Effector Treg and Regulates IL-17A Expression
Source: Front Immunol. 2020 Jan 21;10:3047. doi: 10.3389/fimmu.2019.03047 (PMC6986271; doi:10.3389/fimmu.2019.03047)
Supplement: Table S2 — Target genes used for siRNA interference. [file Table_2.docx]

**Table S2.** siRNA sequences used during the knockdown of *TNFAIP3* gene expression.

| **Code** | **Gene** | **Target Sequence** |
| --- | --- | --- |
| D-001950-01-05 | *Non-targeting siRNA*  *(negative control)* | *UGGUUUACAUGUCGACUAA* |
| D-001970-01-05 | *PPIB*  *(positive control, encoding protein* Cyclophilin B) | *GCCUUAGCUACAGGAGAGA* |
| E-009919-00-0005 | *TNFAIP3* | ***A-009919-13:*** *CUCUUAAAGUUGAUAUCUU*  ***A-009919-14:*** *GCACCAUGUUUGAAGGAUA*  ***A-009919-15:*** *GGAUCAUUCAUCAUUUUAA*  ***A-009919-16:*** *UCAUCGAGUACAGAGAAAA* |
