# Supplementary material for: TNFα-Signaling Modulates the Kinase Activity of Human Effector Treg and Regulates IL-17A Expression
Source: Front Immunol. 2020 Jan 21;10:3047. doi: 10.3389/fimmu.2019.03047 (PMC6986271; doi:10.3389/fimmu.2019.03047)
Supplement: Table S3 — Primers used for RT-qPCR. [file Table_3.docx]

**Table S3.** Primers used during the RT-qPCR

| **Code** | **Gene** | **Encoded Protein** |
| --- | --- | --- |
| 4333768T | *HPRT1* | HPRT1 |
| Hs00174383_m1 | *IL17A* | IL-17A |
| Hs00369400_m1 | *IL17F* | IL-17F |
| Hs00765730_m1 | *NFKB1* | p50 |
| Hs01028901_g1 | *NFKB2* | p52 |
| Hs00355671_g1 | *NFKBIA* | IκBα |
| Hs00168719_m1 | *PPIB* | Cyclophilin B |
| Hs00232399_m1 | *RELB* | RelB |
| Hs01076112_m1 | *RORC* | RORɣt |
| Hs00234713_m1 | *TNFAIP3* | A20 |
| Hs01042313_m1 | *TNFRSF1A* | TNFR1 |
| Hs00961750_m1 | *TNFRSF1B* | TNFR2 |
